# Satisfaction With Life, Mental Health Problems and Potential Alcohol-Related Problems Among Norwegian University Students

**DOI:** 10.3389/fpsyt.2021.578180

**Published:** 2021-02-09

**Authors:** Pia Jensen, Ellen Haug, Børge Sivertsen, Jens Christoffer Skogen

**Affiliations:** ^1^Department of Health Promotion and Development, University of Bergen, Bergen, Norway; ^2^NLA University College, Bergen, Norway; ^3^Department of Health Promotion, Norwegian Institute of Public Health, Bergen, Norway; ^4^Department of Research and Innovation, Helse Fonna HF, Haugesund, Norway; ^5^Department of Mental Health, Norwegian University of Science and Technology, Trondheim, Norway; ^6^Alcohol & Drug Research Western Norway, Stavanger University Hospital, Stavanger, Norway; ^7^Department of Public Health, Faculty of Health Sciences, University of Stavanger, Stavanger, Norway

**Keywords:** population-based study, university students, college students, mental health problems, satisfaction with life, potential alcohol-related problems

## Abstract

**Objective:** Recent studies have shown that today's college students more than ever are struggling with mental health and alcohol problems. While poor satisfaction with life and mental health problems have been linked to higher alcohol consumption, there is still a lack of studies examining in detail the shape and nature of the relationship between mental health and alcohol consumption.

**Aim:** To investigate the associations between satisfaction with life, mental health problems and potential alcohol-related problems among Norwegian university students. The shape of the associations was also examined.

**Methods:** Data were drawn from a 2018 national survey of students in higher education in Norway (the SHoT-study). Associations between satisfaction with life, mental health problems and potential alcohol-related problems (AUDIT; risky and harmful alcohol use) were investigated using logistic regression. Both crude models and models adjusted for age, gender and marital status were conducted. To investigate the shape of the associations, logistic regression with quadric and cubic terms was tested.

**Results:** Decreased satisfaction with life and increased mental health problems were associated with potential alcohol-related problems. For satisfaction with life, a curvilinear association with risky alcohol use and a linear association with harmful alcohol use was identified. For mental health problems, curvilinear associations were found for both risky and harmful alcohol use.

**Conclusion:** Many students report potential alcohol-related problems. Students with harmful alcohol use seem to be more at risk of reduced satisfaction with life and increased mental health problems than students with risky alcohol use. Educational institutions may be an ideal setting for raising awareness of mental health issues and responsible alcohol consumption among students. The present study contributes with important information about the shape of the associations between satisfaction with life, mental health problems and potential alcohol-related problems in the student population.

## Introduction

College and university students represent a special group in the society, with unique characteristics and challenges generally not found elsewhere (1, 2). For instance, the transition to student life typically implies greater independence, and involves formation of new friendships and identities (3). It is also a period of risky behaviors and increased experimentation with different substances (4, 5). This may explain why enrollment in higher education often is associated with increased alcohol consumption, that potentially can be classified as harmful (6, 7). When looking at the general population, the high alcohol use among students may also be explained by the fact that there is a peak in alcohol consumption in the age period between 18 and 29 years (8, 9). In younger age groups, some studies have also found that high and harmful alcohol use increase more sharply with lower socioeconomic status in countries such as England (10) and Norway (11). In contrast, a study with undergraduate students from Brazil found that students with higher monthly family income reported higher alcohol use than students with lower monthly family income (12). The fact that people with higher socioeconomic status are consuming more alcohol, but the people with lower socioeconomic status are experiencing more alcohol-related problems is called the “alcohol-harm paradox” (13).

According to multiple studies, alcohol is the most commonly used substance among students, compared to drugs and tobacco (5, 14–16). Although students consider alcohol to be a positive aspect of the student life (17), alcohol use has several negative health consequences. For instance, prolonged heavy drinking increases the risk of certain types of cancer, heart and vascular diseases, liver damage, mental disorders, suicide attempts and suicide (18). While these problems may take time to develop, acute intoxication increases the risk for accidents, risk-taking and intentional and unintentional injuries (19, 20).

Extensive research indicates the presence of a relationship between alcohol use and mental health problems (21–25). This association may be particularly relevant to investigate among students since they are in an age-period where high-prevalence mental disorders (depression, and anxiety) emerge (26). Former studies have typically found that increased levels of mental health problems lead to increased alcohol consumption (25) or reversed (27). A particular relevant study was done by Sæther et al. (17), who used data from the Norwegian SHoT 2014 survey. They found that students who reported risky alcohol use (AUDIT 8-17) had slightly reduced life satisfaction and more mental health problems compared with the students who reported low-risk alcohol use. In addition, the students who reported harmful alcohol use (AUDIT ≥18) had both low life satisfaction and more mental health problems compared with the other students.

How gender affects the association between mental health problems and alcohol consumption among college and university students is not clear. Some studies have found that the association is stronger among female students (2, 28), while Pedrelli et al. (29) for instance found that worse symptoms of depression was associated with increased daily alcohol use among male students, but not among female students.

Further evidence suggests that the association between mental health problems and alcohol use may not be linear (30). For instance, Skogen et al. (24) found a *U*-shaped relationship between alcohol consumption and the risk of depression and anxiety among Norwegian adults, while Caldwell et al. (31) found a *J*-shaped association between alcohol consumption and depression among Australian young adults. In addition, Peltzer and Pengpid (32) found an inverse *U*-shaped association between alcohol consumption and depression among students. Skogen et al. (33) found neither a *U*-shaped nor a *J*-shaped relation between alcohol use and mental health problems among adolescents, which indicate the existence of other forms of associations across different age groups. It is unclear if a non-linear association can be identified across all age groups (23), and an examination of the association among university and college students is warranted as it may have practical implications for the development of more tailored interventions aimed at reducing alcohol use among students.

As mentioned above, severe mental health problems and physical consequences associated with heavy drinking take time to develop and may be less likely to affect students (17). However, students may experience immediate adverse events, such as injuries and accidents (20), and reduced academic performance (34). Such events might affect an individual's satisfaction with life, and some studies have found an association between alcohol-related problems and diminished life satisfaction among college and university students (35–38). Generally, there has been less research investigating the relationship between positive measures of well-being, such as satisfaction with life, and alcohol consumption. This points to a need for more research.

Massin and Kopp (39) examined the shape of the association between life satisfaction and alcohol use among adults. They found an inverse *U*-shaped relation among women and inverse *J*-shaped relation among men in the crude models. Male heavy drinkers reported lower life satisfaction than abstainers, and both female heavy drinkers and abstainers reported lower life satisfaction than moderate drinkers. In the adjusted analyses, the humped-shaped curve became increasingly flattened in all samples. Among women, the linear and quadric models became non-significant. Among men and in the full sample, only the quadric model remained significant. To our knowledge, no research has investigated the shape of this association in the student population.

Based on the above considerations, the present study aimed to investigate how satisfaction with life and mental health problems are associated with potential alcohol-related problems among Norwegian college and university students. The article focused on alcohol, as this is by far the most used substance among Norwegian students (16). Since there are inconsistent results of the shape of the association between satisfaction with life, mental health problems and alcohol use in the general population, the present study also aimed to investigate the shape of the association among students enrolled in higher education.

## Materials and Methods

### Procedures and Sample

The Students' Health and Well-being Study, the SHoT study, is a large cross-sectional questionnaire survey conducted every 4 years targeting all Norwegian students enrolled at either a university or college (40). Details of the SHoT study have been published elsewhere (40). The present study used data from the most recent wave collected from February to April 2018 (the SHoT-2018 study), and included all full-time students (both in Norway and abroad) between the ages of 18 and 35 with Norwegian citizenship. An email with a link to the questionnaire was sent to the 162 512 students who fulfilled the inclusion criteria, of whom 50 054 students completed the questionnaire, yielding a response rate of 30.8% (40).

All participants were asked to report their gender and age. In comparison with all invited students [58.1% women (*n* = 93 267) and 41.9% men (*n* = 67 558)], the SHoT 2018 sample included 69.1% women and 30.9% men (40). The mean age in the study sample was 23.2 (SD = 3.3) years, and the age distribution of the participating students was very similar to the age distribution of the invited students (40).

### Ethics

The Regional Committee for Medical and Health Research Ethics in Western Norway approved the SHoT 2018 Study (no. 2017/1176). Informed consent was obtained electronically after the participants had received a detailed introduction to the study.

### Measurement Instruments

#### Dependent Variable: Potential Alcohol-Related Problems (AUDIT)

In the present study, a translated Norwegian version of the Alcohol Use Disorders Identification Test (AUDIT) was used to measure potential alcohol-related problems, with a timeframe of the last 12 months. AUDIT is a widely used instrument to identify individuals with risky and harmful patterns of alcohol use developed by the World Health Organization (41). AUDIT consists of 10 items, and each item has response options that can be scored from 0–4, creating a sum score ranging from 0–40. As recommended, a total score of ≥8 was used as a cut-off for potential alcohol-related problems (41–43). Further, a score between 8 and 15 indicated risky alcohol use and a score of 16 and above indicated harmful alcohol use. In the descriptive analyses, potential alcohol-related problems were indicated by a dichotomous variable, which separated the students scoring below eight (low-risk alcohol use, coded 0) and the students scoring eight or above (potential alcohol-related problems, coded 1). In the logistic regression analyses, potential alcohol-related problems were further divided into risky alcohol use (AUDIT 8-15, coded 1) and harmful alcohol use (AUDIT ≥16, coded 1) (see Table 1). The same reference group was therefore used in all the dependent variables (AUDIT <8, coded 0). In the present study, AUDIT showed acceptable internal consistency with a Cronbach's alpha of 0.75. In order to describe the drinking pattern of the sample in further detail, three dichotomous variables based on the three first AUDIT-questions were created. AUDIT-question #1 is “How often do you have a drink containing alcohol?,” with “never,” “monthly or less,” “2–4 times a month,” “2–3 times a week” and “4 or more times a week” as response options. For AUDIT#1, we differentiated between abstainers and drinkers. AUDIT-question #2 is “How many standard drinks [units] containing alcohol do you have on a typical day when drinking?,” with the following response options “1 or 2,” “3 or 4,” “5 or 6,” “7 or 9,” “10 or more.” For AUDIT#2, we differentiated between <7 units and 7 or more units. AUDIT-question #3 is “How often do you have six or more drinks on one occasion?,” with “Never,” “Less than monthly,” “Monthly,” “Weekly” and “Daily or almost daily” as response options. For AUDIT#3 we differentiated between drinking six or more units less than monthly and drinking six or units or more monthly or more. AUDIT and potential alcohol-related problems across pertinent characteristics in SHoT are presented in a separate publication (44).

**Table 1:** Overall characteristics of sample across gender.

	***N***		**Overall**	**Female**	**Male**	***p*-value**
Age (SD)^a^	49,157^d^		23.2 (3.3)	23.1 (3.3)	23.5 (3.3)	<0.001
Gender^b^	49,836^d^		NA	69.2%	30.8%	NA
Marital status^b^	49,764^d^					<0.001
		Single (%)	50.0	47.2	56.0	
		Relationship (%)	23.8	24.0	23.3	
		Cohabitant (%)	22.8	25.1	17.7	
		Married (%)	3.4	3.7	3.0	
Semesters studied (IQR)^c^	49,673^d^		4 (2–7)	4 (2–7)	5 (2–8)	=0.062
Mean AUDIT score (SD)^a^	49,690^d^		7.3 (4.7)	6.8 (4.5)	8.2 (5.1)	<0.001
Abstainers last 12 months (AUDIT#1)^b^		Yes (%)	7.9	8.1	7.6	<0.001
Usually drinks seven or more units (AUDIT#2)^b^		Yes (%)	21.7	15.9	35.1	<0.001
Six units monthly or more in one sitting (AUDIT#3)^b^		Yes (%)	40.5	36.0	50.8	<0.001
Satisfaction with life (SD)^a^	48,418^d^		22.0 (6.7)	21.9 (6.7)	22.1 (6.9)	=0.005
Mental health problems (SD)^a^	49,730^d^		1.7 (0.6)	1.8 (0.6)	1.5 (0.5)	<0.001

*NA, Not applicable; SD, Standard deviation; IQR, Interquartile range*.

a *Independent sample t-test*.

b *Chi-square test*.

c *Two-sample Wilcoxon rank-sum (Mann-Whitney) test*.

d *Pairwise-deletion based on valid responses on gender variable*.

#### Independent Variable: Satisfaction With Life (SWLS)

Satisfaction with life was assessed using a translated Norwegian version of the satisfaction with life scale (SWLS) (45). SWLS consists of five items measuring global life satisfaction. It is designed to measure global cognitive judgement of one's life satisfaction, and not a measure of affectivity *per se*. Participants indicate their agreement with each item using a seven-point scale, where one indicated “strongly disagree” and seven “strongly agree.” Based on the participants responses, the possible range of scores is from 5 (low satisfaction) to 35 (high satisfaction) (45). SWLS showed a good internal consistency in the present study, indicated by a Cronbach's alpha of 0.89.

#### Independent Variable: Mental Health Problems (HSCL-25)

Mental health problems were assessed using a translated Norwegian version of the 25 item Hopkins Symptom Checklist (HSCL-25) (46). HSCL-25 contains questions related to symptoms of anxiety and depression. According to Skogen et al. (47), who investigated the factor structure of the scale in a student population (using data from SHoT 2014), a uni-dimensional model is most appropriate. This finding was reasserted in SHoT 2018 [see (40)]. Participants are asked to assess to what degree different symptoms have bothered them during the past 2 weeks. Each item in the scale is scored on a four-point scale, where one indicated “not bothered” and four “extremely bothered.” The mean summed score ranges from 1 (low symptom load) to 4 (high symptom load). In the present study the Cronbach's alpha was 0.94 and indicated excellent internal consistency. In Table 1 we present mean HSCL-25 score as both a continuous and a dichotomous measure [using cut-off ≥2.00; (48)]. For all other analyses we use HSCL-25 as continuous measure in order to retain maximum level of information.

#### Covariates

In the present study age, gender and marital status were included as potential confounders. Marital status was recorded across four categories, distinguishing between single, girlfriend/boyfriend, cohabitant and married. In addition, we present the number of semesters studied in the first descriptive table.

### Statistical Analysis

All data analyses were conducted using Stata 15 (49). Missing data were deleted pairwise. First, independent samples *t*-tests, Wilcoxon rank-sum tests, and **χ**^2^-tests were used to examine gender-differences across the demographic characteristics age and marital status, and semesters studied, mean AUDIT score, proportion abstainers and consumption patterns, as well as satisfaction with life and mental health problems (Table 1). Mean and median scores and proportions were calculated for the total study population, as well-across gender.

Second, independent samples *t*-tests and **χ**^2^-tests were used to examine differences on the demographic characteristics age, gender and marital status, satisfaction with life and mental health problems (continuous and dichotomous) across those scoring below and above the cut-off for potential alcohol-related problems (≥8) (Table 2). Mean scores and proportions were calculated and compared between the two AUDIT groups. Third, separate logistic regression models with risky alcohol use or harmful alcohol use as dependent variable were estimated for satisfaction with life and mental health problems as independent continuous variables (Tables 3, 4). To allow for direct comparison of effect sizes, both independent variables were standardized (*Z*-scored) before they were entered into the logistic regression models. Crude associations as well as associations adjusted for age, gender and marital status were estimated for satisfaction with life and mental health problems. Fourth, for the analysis of possible curvilinear associations, separate logistic regression models with quadratic and cubic terms were estimated for satisfaction with life and mental health problems. The analyses operated with either risky alcohol use or harmful alcohol use as the dependent variable. The analyses were estimated as a crude model and as a model adjusted for age, gender and marital status. In the logistic regression models the odds ratios were used as the effect measure along with the 95% confidence intervals (95% CI). Finally, likelihood-ratio tests were used to compare models (linear, quadratic or cubic) (Tables 5, 6) (50). The associations between satisfaction with life and potential alcohol-related problems were estimated. Linear and quadratic model were compared, followed by the cubic vs. the quadratic (50). This was conducted for both risky alcohol use and harmful alcohol use as dependent variables. The models that best fitted the data when considering the results of the likelihood-ratio tests, as well as substantive interpretation and parsimony, were used to assess potential gender-interactions between satisfaction with life and potential alcohol-related problems. Specifically, a likelihood-ratio test comparing the best fitting model with and without an interaction term (satisfaction with life × gender) was estimated for both risky alcohol use and harmful alcohol use as dependent variables (Table 7). Gender-stratified results were presented if the likelihood-ratio test indicated a statistical significant gender-interaction. The same procedure was repeated for the association with mental health problems. Additional gender-specific results of the main analyses is available in the Appendix. The number of individuals with missing data on AUDIT, SWLS and HSCL-25 was 168 (0.3%), 1 446 (2.9%) and 132 (0.3%), respectively.

**Table 2:** Characteristics of the sample across AUDIT scores <8 vs. ≥8.

	***N***		**AUDIT <8 (56.3%)**	**AUDIT≥8 (43.7%)**	***p*-value**
Age (SD)^a^	49,202^c^		23.5 (3.6)	22.9 (2.8)	<0.001
Gender^b^	49,690^c^				<0.001
		Female (%)	60.3	39.7	
		Male (%)	47.5	52.5	
Marital status^b^	49,812^c^				<0.001
		Single (%)	43.8	58.0	
		Relationship (%)	23.7	23.8	
		Cohabitant (%)	27.1	17.2	
		Married (%)	5.4	0.9	
Satisfaction with life (SD)^a^	48,571^c^		22.3 (6.8)	21.5 (6.6)	<0.001
Mental health problems (SD)^a^	49,811^c^		1.7 (0.6)	1.8 (0.6)	<0.001
Mental health problems (cutoff ≥2.00)^b^	49,811^c^				<0.001
		Below cut-off (%)	72.9	69.5	
		Above cut-off (%)	27.1	30.5	

*SD, Standard deviation*.

a *Independent sample t-test*.

b *Chi-square test*.

c *Pairwise-deletion based on valid responses on AUDIT score variable*.

**Table 3:** Crude and adjusted association between satisfaction with life (SWLS) and potential alcohol-related problems (AUDIT≥8).

	AUDIT (≥8 <16; 38.2%)^*^		AUDIT (≥16; 5.5%)^*^	
	**Odds ratio (CI 95%)**	***p*-value**	**Odds ratio (CI 95%)**	***p*-value**
Satisfaction with life, crude	0.92 (0.90–0.94)	<0.001	0.67 (0.65–0.70)	<0.001
Satisfaction with life, adjusted for age	0.91 (0.89–0.93)	<0.001	0.67 (0.64–0.70)	<0.001
Satisfaction with life, adjusted for gender	0.91 (0.90–0.93)	<0.001	0.68 (0.65–0.70)	<0.001
Satisfaction with life, adjusted for marital status	0.97 (0.95–0.98)	<0.001	0.73 (0.70–0.76)	<0.001
Satisfaction with life, fully adjusted	0.95 (0.93–0.97)	<0.001	0.72 (0.69–0.75)	<0.001

*Logistic regression models. N = 47,686*.

* *AUDIT <8 represents the low risk group*.

*SWLS was standardized (Z-scored) in the logistic regression models*.

**Table 4:** Crude and adjusted association between mental health problems (HSCL-25) and potential alcohol-related problems (AUDIT≥8).

	AUDIT (≥8 <16; 38.2%)^*^		AUDIT (≥16; 5.5%)^*^	
	**Odds ratio (CI 95%)**	***p*-value**	**Odds ratio (CI 95%)**	***p*-value**
Mental health problems, crude	1.04 (1.02–1.06)	<0.001	1.46 (1.41–1.52)	<0.001
Mental health problems, adjusted for age	1.04 (1.02–1.06)	<0.001	1.46 (1.41–2.51)	<0.001
Mental health problems, adjusted for gender	1.11 (1.09–1.13)	<0.001	1.67 (1.61–1.73)	<0.001
Mental health problems, adjusted for marital status	1.04 (1.02–1.06)	<0.001	1.45 (1.40–1.51)	<0.001
Mental health problems, fully adjusted	1.10 (1.08–1.12)	<0.001	1.64 (1.58–1.70)	<0.001

*Logistic regression models. N = 48,886*.

* *AUDIT <8 represents the low risk group*.

*HSCL-25 was standardized (Z-scored) in the logistic regression models*.

**Table 5:** Likelihood-ratio tests for the associations between satisfaction with life, mental health problems and potential alcohol-related problems.

	**AUDIT (≥8 <16; 38.2%)**		**AUDIT (≥16; 5.5%)**	
	**Likelihood ratio**	***P*-value**	**Likelihood ratio**	***P*-value**
Satisfaction with life, fully adjusted	Quadric vs. linear	<0.001	Quadric vs. linear	0.790
	Cubic vs. quadric	0.557	Cubic vs. quadric	0.150
Mental health problems, fully adjusted	Quadric vs. linear	<0.001	Quadric vs. linear	<0.001
	Cubic vs. quadric	0.001	Cubic vs. quadric	0.134

**Table 6:** Likelihood-ratio test for the association between mental health problems and risky alcohol use after the removal of 2.5% of the respondents on each end of the HSCL-25 scale.

	**AUDIT (≥8 <16; 38.2%)**	
	**Likelihood-ratio**	***P*-value**
Mental health problems, fully adjusted	Quadric vs. linear	<0.001
	Cubic vs. quadric	0.139

**Table 7:** Likelihood-ratio tests for a potential gender-interaction in associations between satisfaction with life, mental health problems and potential alcohol-related problems.

		**AUDIT (≥8 <16; 38.2%)**	**AUDIT (≥16; 5.5%)**
	**Likelihood ratio**	***P*-value**	***P*-value**
Satisfaction with life, adjusted for age and marital status	Gender-interaction vs. no gender interaction	<0.001	<0.001
Mental health problems, adjusted for age and marital status	Gender-interaction vs. no gender interaction	<0.001	=0.035

## Results

Table 1 presents relevant descriptive statistics of the overall sample, and across gender. The overall age was 23.2 years [Standard deviation (SD) = 3.3], with a slightly higher age among males compared to females. Fifty percent of the overall sample reported being single, and more females compared to males reported being in a relationship. The median number of semesters studied was four (interquartile range 2–7), with no gender differences. The mean overall AUDIT-score was 7.3 (SD = 4.7), with higher scores among males compared to females. In relation to abstaining, 7.9% of the overall sample reported that they had not consumed alcohol the last 12 months, with a somewhat higher proportion among females compared to males. Over 1/5 (21.7%) of the overall sample reported usually drinking seven or more units on a typical drinking day, with marked differences between males (35.1%) and females (15.9%). Over half (50.8%) of the males compared to 36.0% of females reported drinking six units or more on one drinking occasion monthly or more often, while the overall proportion was 40.5%. The overall mean level of satisfaction with life (SWLS) was 22.0 (SD = 6.7), with slightly higher mean among males compared to females. The overall mean level of mental health problems was 1.7 (SD = 0.6), with higher levels of reported problems among females compared to males. The correlation between HSCL mean score and the mean summed score on SWLS was −0.56.

Table 2 presents the main characteristics of those scoring below and above the cut-off for potential alcohol-related problems (AUDIT ≥8). Individuals above the cut-off reported lower levels of satisfaction with life (Mean (M) = 21.5, SD = 6.6) and higher levels of mental health problems (*M* = 1.8, SD = 0.6), compared to the low risk group. For the demographic variables, it was also found that the individuals above the threshold for potential alcohol-related problems were somewhat younger (*M* = 22.9, SD = 2.8 vs. *M* = 23.5, SD = 3.6), more likely to be male (52.5 vs. 39.7%) and single compared to the low risk group.

**Figure 1:**
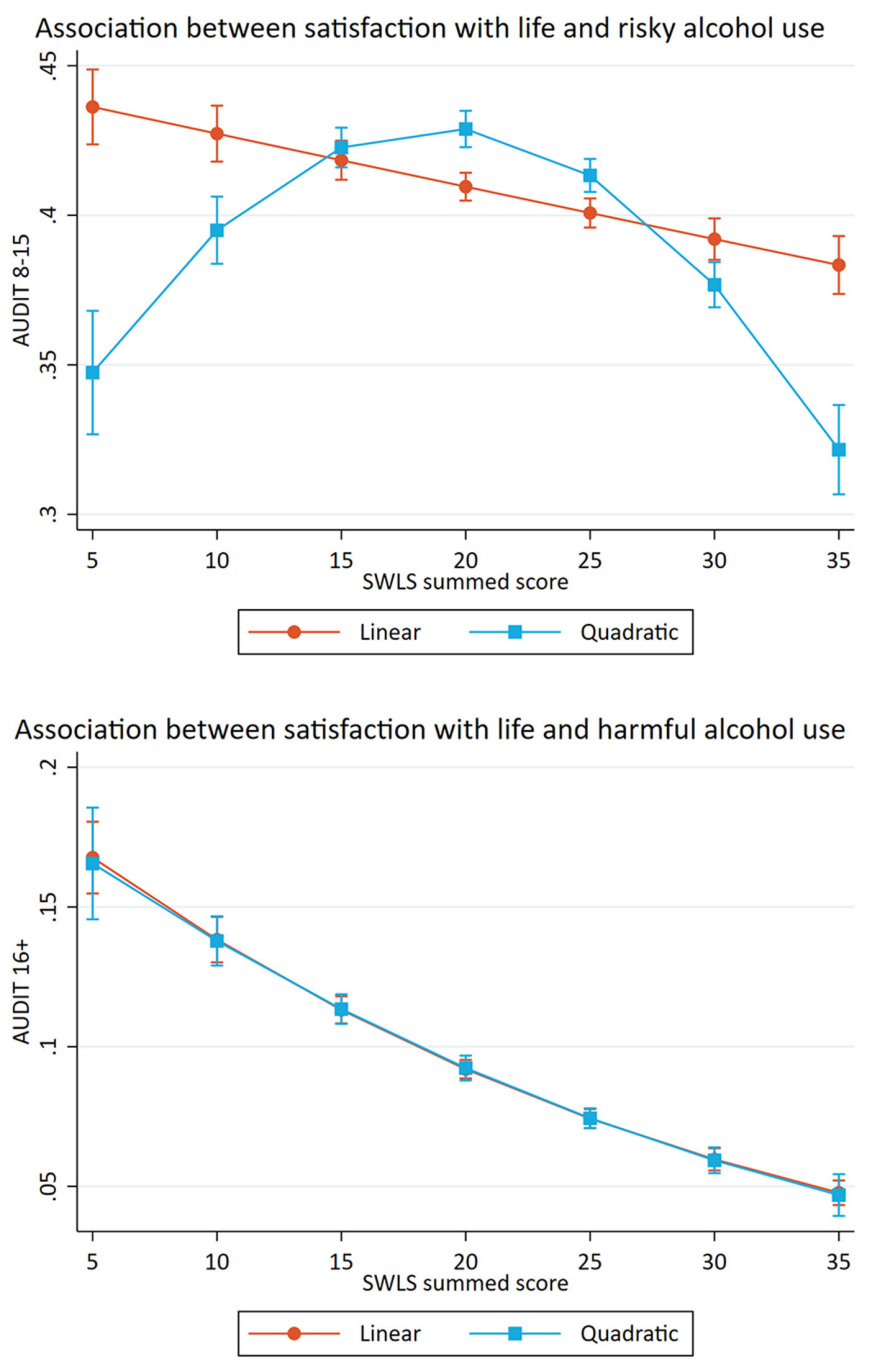
Associations between satisfaction with life and potential alcohol-related problems. Predicted probabilities of alcohol-related problems (The Alcohol Use Disorders Identification test; AUDIT) across summed scores on satisfaction with life scale (SWLS). Error bars represent 95% confidence intervals.

Table 3 shows the crude and adjusted association between satisfaction with life (SWLS) and potential alcohol-related problems. For the association between satisfaction with life and risky alcohol use, the crude model showed a significant association. Separate adjustments for sociodemographics (age, gender and marital status), yielded only a marginal change in the OR after controlling for marital status (see Table 3). The fully adjusted model indicated that a one standardized (*Z*-scored) unit increase in satisfaction with life was linearly associated with decreased odds of reporting risky alcohol use (Odds ratio (OR): 0.95, *p* < 0.001). A significant association between satisfaction with life and harmful alcohol was also found in the crude model. Separate adjustments for sociodemographics, yielded only a marginal change in the OR after adjusting for marital status (see Table 3). The fully adjusted model indicated that a one standardized unit increase in satisfaction with life was associated with decreased odds of reporting harmful alcohol use (OR: 0.72, *p* < 0.001). Estimated probabilities for associations between life satisfaction and potential alcohol-related problems are presented in Figures 1, **4**.

**Figure 2:**
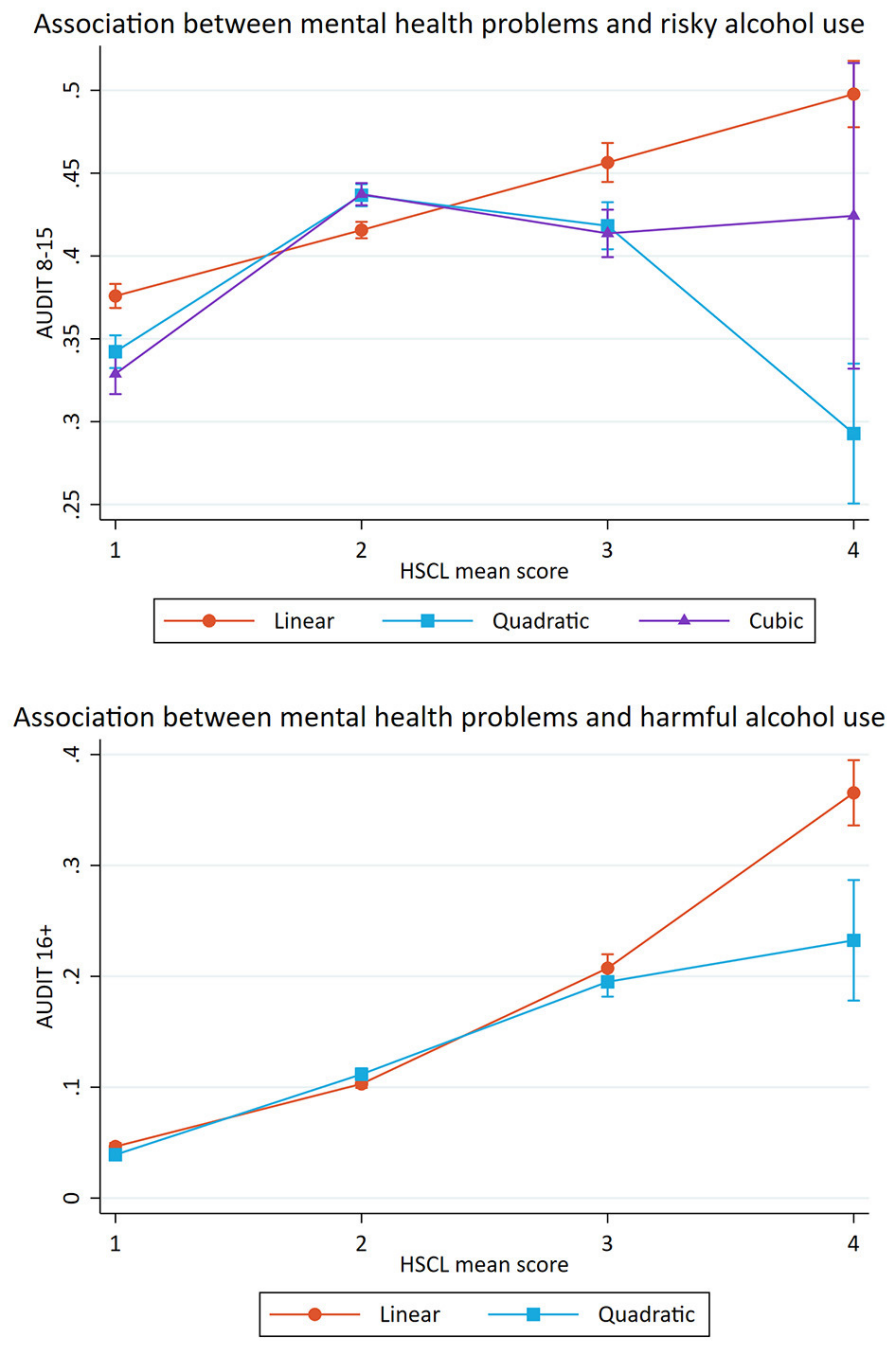
Association between mental health problems and potential alcohol-related problems. Predicted probabilities of alcohol-related problems (The Alcohol Use Disorders Identification test; AUDIT) across mean scores on Hopkins Symptom Checklist (HSCL). Error bars represent 95% confidence intervals.

**Figure 3:**
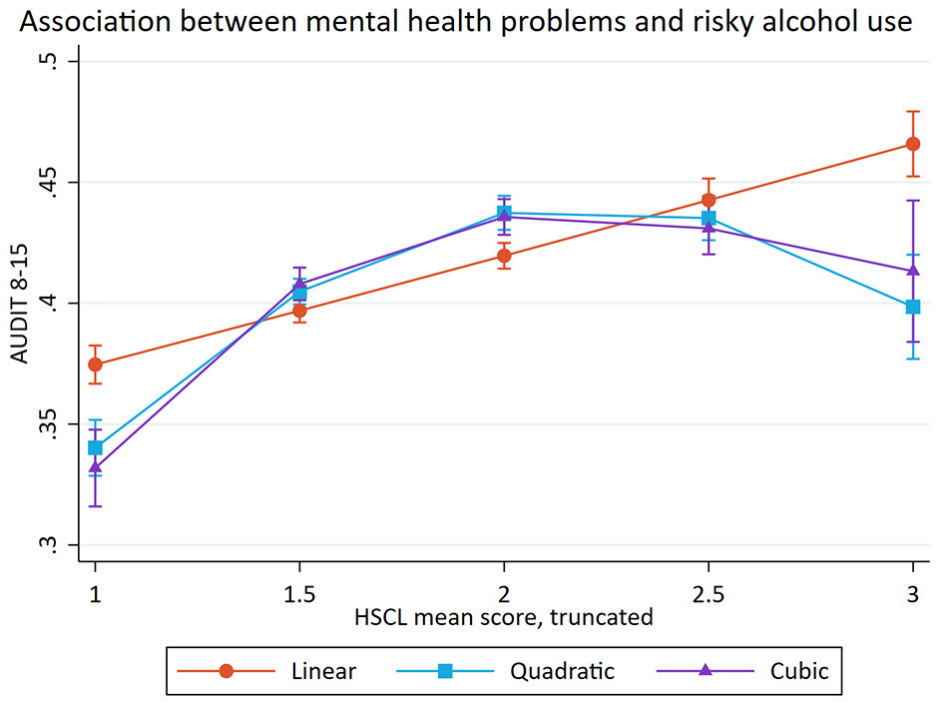
Association between mental health problems and risky alcohol use (after the removal of 2.5% in each end of HSCL-25 scale). Predicted probabilities of alcohol-related problems (The Alcohol Use Disorders Identification test; AUDIT) across mean scores on Hopkins Symptom Checklist (HSCL). Error bars represent 95% confidence intervals.

Table 4 shows the crude and adjusted association between mental health problems (HSCL-25) and potential alcohol-related problems. The crude model showed a significant association between mental health problems and risky alcohol use. After separate adjustments for sociodemographics (age, gender and marital status), a small change in the OR after controlling for gender was observed (see Table 4). The fully adjusted model indicated that a one standardized unit increase in mental health problems was associated with increased odds of reporting risky alcohol use (OR: 1.10, *p* < 0.001). A significant association between mental health problems and harmful alcohol was also found in the crude model. Separate adjustments for sociodemographics resulted in a considerable change in the OR when adjusting for gender (see Table 4). The fully adjusted model indicated that a one standardized unit increase in mental health problems was associated with increased odds of reporting harmful alcohol use (OR: 1.64, *p* < 0.001). Estimated probabilities for associations between mental health problems and potential alcohol-related problems are presented in Figures 2, 3, **5**.

Table 5 shows the separate likelihood-ratio tests for the fully adjusted associations between satisfaction with life, mental health problems and potential alcohol-related problems. The likelihood-ratio tests include the results from the fully adjusted logistic regression models with linear vs. quadric and cubic terms. In the present study, the linear model was the simplest model, followed by the curvilinear model and last the cubic model which represented the most complex model. The *p*-value indicates whether a more complex model better fits the data. For the association between satisfaction with life and potential alcohol-related problems, the results indicated that the quadric model was the best fit for the association with risky alcohol use and that the linear model was the best fit for the association with harmful alcohol use (see Figure 1).

Further, the likelihood-ratio tests for the association between mental health problems and potential alcohol-related problems indicated that the cubic model was the best fit for the association with risky alcohol use and that the quadric model was the best fit for the association with harmful alcohol use (see Figure 2).

For the cubic model, the confidence interval of scoring four on the HSCL-25 was very large, indicating low precision in the estimate. As a result, a *post-hoc* analysis investigating if the small portion of the study sample (together 5% of the sample, 2.5% on each end of the HSCL-25 scale) who scored the most extreme values one and four affected the shape of the association was performed. Table 6 shows the likelihood-ratio test where 2.5% of the responses were removed. The model with truncated HSCL-25 scores showed that the quadric model best explained the association between mental health problems and risky alcohol use (see Figure 3), and we chose to retain the quadratic model instead of the cubic model.

**Figure 4:**
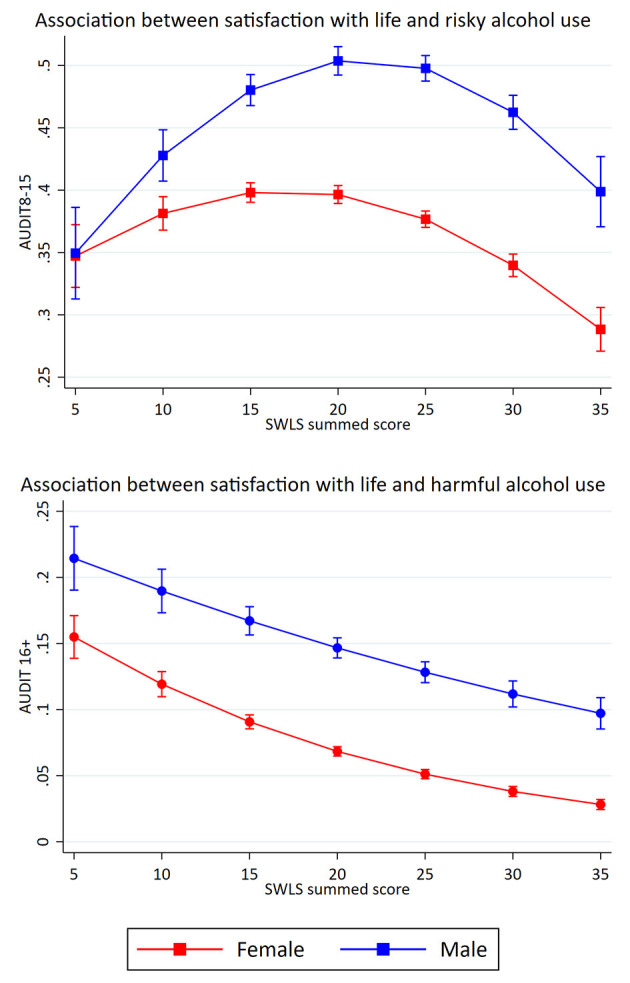
Gender-specific associations between satisfaction with life and potential alcohol-related problems. Retained models. Predicted probabilities of alcohol-related problems (The Alcohol Use Disorders Identification test; AUDIT) across summed scores on satisfaction with life scale (SWLS). Error bars represent 95% confidence intervals.

**Figure 5:**
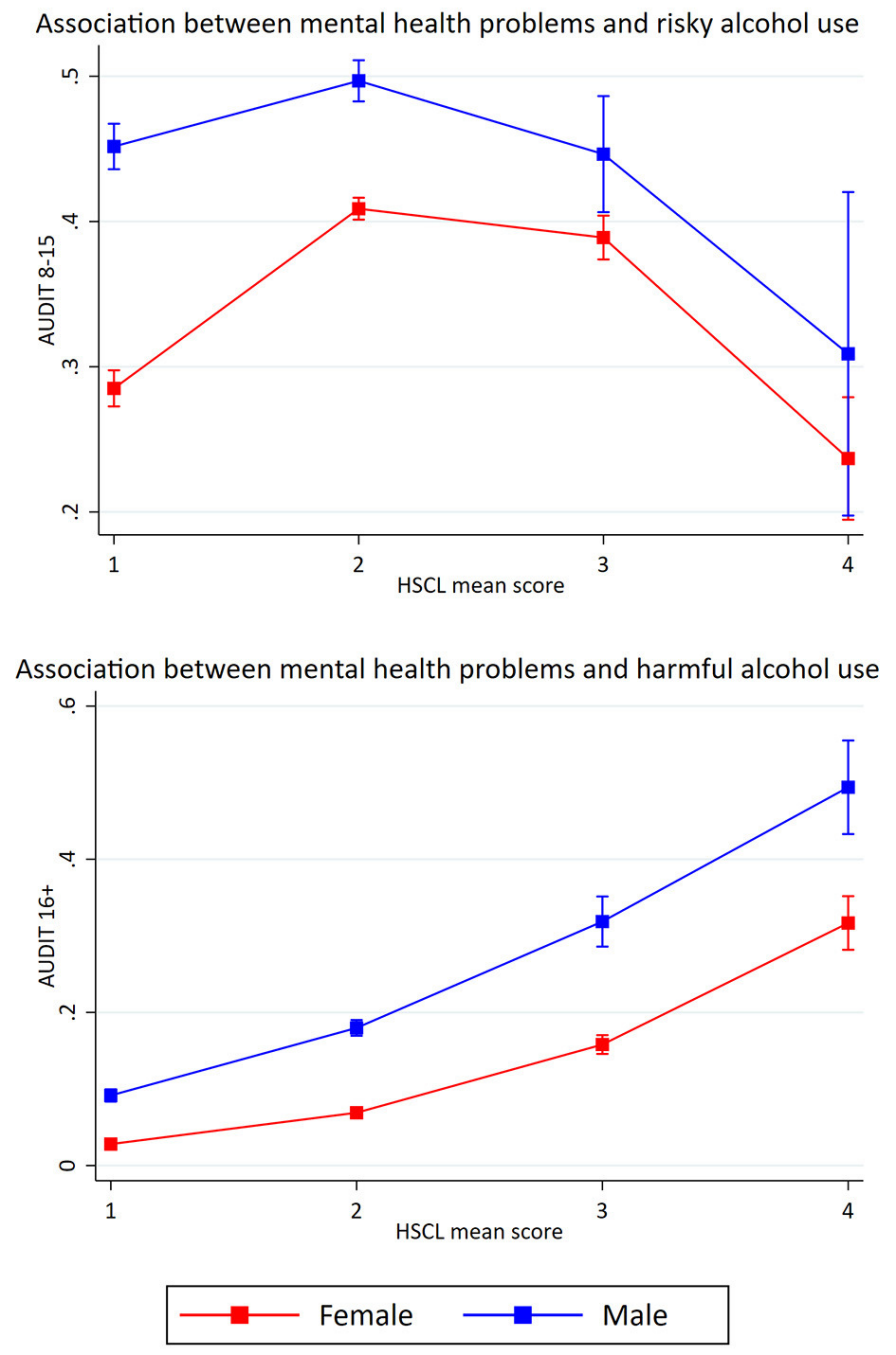
Gender-specific associations between mental health problems and potential alcohol-related problems. Retained models. Predicted probabilities of alcohol-related problems (The Alcohol Use Disorders Identification test; AUDIT) across mean scores on Hopkins Symptom Checklist (HSCL). Error bars represent 95% confidence intervals.

In the retained models, we investigated potential gender-interactions (Table 7). For each of the retained models, the likelihood-ratio tests indicated that models that included a gender-interaction fitted the data better (all *p*-values <0.05). Therefore, gender-stratified results of all the retained models are presented (Figures 4, 5). For satisfaction with life, the curvature was more pronounced among males compared to females, while there was a slight curvature for females and not for males in relation to harmful alcohol use (Figure 4). A likelihood-ratio test comparing a linear vs. a quadratic models for females only, did, however not indicate that a quadratic model was better for females (*p* = 0.558). For mental health problems in relation to risky alcohol use (Figure 5), the curvature was more pronounced among females, while the overall relationship with harmful alcohol use were similar across gender, although the increase was more pronounced among males with higher HSCL mean scores.

## Discussion

### Summary of Main Results

This national study of college and university students in Norway investigated the associations between satisfaction with life, mental health problems and potential alcohol-related problems. Results from the logistic regression models support the existence of an association between (1) satisfaction with life and potential alcohol-related problems and (2) mental health problems and potential alcohol-related problems. In the adjusted regression models, it was found that one standardized unit increase in satisfaction with life reduced the odds of reporting both risky and harmful alcohol use. The opposite association was found between mental health problems and potential alcohol-related problems, where one standardized unit increase in mental health problems increased the odds of reporting both risky and harmful alcohol use.

The present study also investigated the shape of the associations between satisfaction with life, mental health problems and potential alcohol-related problems. For satisfaction with life, the results indicated a curvilinear association with risky alcohol use and a linear association with harmful alcohol use. For mental health problems, the results indicated curvilinear associations with both risky and harmful alcohol use. Taken together, the different results related to risky and harmful alcohol use showed the usefulness of dividing potential alcohol-related problems in to two different subcategories.

### Association Between Satisfaction With Life and Potential Alcohol-Related Problems

The observed association between satisfaction with life and potential alcohol-related problems indicated that increased levels of satisfaction with life reduced the risk of reporting potential alcohol-related problems among students. The findings are in line with other studies that have investigated the relation between alcohol use and life satisfaction, with life satisfaction as the dependent variable. Blank et al. (37) found, after adjusting for personality, social and individual variables, that female students who reported a hazardous alcohol use (AUDIT-C score ≥5) had lower well-being compared to abstaining participants. Among male students, it was found that those who reported a hazardous (AUDIT-C score ≥7) and moderate alcohol use (AUDIT-C score 1-6) had lower mental well-being compared to abstaining participants. Further, Mohamed and Ajmal (38) found after adjusting for age and gender (among others), that hazardous drinkers (as indicated by higher AUDIT-C values) reported lower quality of life.

#### The Shape of the Association Between Satisfaction With Life and Potential Alcohol-Related Problems

The shape of the association between satisfaction with life and potential alcohol-related problems were different for the two subcategories. There was an inverse *U*-shaped association between satisfaction with life and risky alcohol use, where the students with a moderate life satisfaction had the highest probability of belonging to this group. The group with risky alcohol use represented a considerable proportion of the students (38.2%), which supports previous studies that have found that this drinking pattern is common among Norwegian students (17, 51). There was also an inverse *U*-shaped association between satisfaction with life and risky alcohol use for both female and male students, although the curvature was more pronounced among males. The finding of an inverse *U*-shaped relationship among the full sample is in line with Massin and Kopp (39), who also investigated the association between alcohol consumption and life satisfaction. Contrary to the present study, they found that heavy drinkers and abstainers reported lower life satisfaction in the full sample. Massin and Kopp also found an inverse *U*-shaped association among women, but contrary to our findings, they found an inverse *J*-shaped association among males. The differences may be due to the fact that Massin and Kopp (39) measured alcohol consumption, and the present study used AUDIT as a measure of risky alcohol use. In addition, Massin and Kopp (39) measured life satisfaction by only one question, and the current study used SWLS. Since Massin and Kopp (39) also had a sample with a broader age span, the result from the present study may indicate that period in life is meaningful when investigating the association between satisfaction with life and alcohol use.

In contrast, there was a linear association between satisfaction with life and harmful alcohol use. This was found in all samples (full sample, female and male students). The results implied that the students with low satisfaction with life had the highest probability of belonging to the group. The risk of reporting a harmful alcohol use gradually decreased, by increasing levels of satisfaction with life. The group with a harmful alcohol use represented only a small portion of the students (5.5%), which may imply that those students have a drinking pattern that is statistically deviant—even in the student population. This drinking pattern may be interpreted as problem drinking, where alcohol often is used as a coping strategy (52). In contrast, the findings of a large proportion of students with risky alcohol use may indicate that this drinking pattern reflects social drinking among students.

The association between satisfaction with life and potential alcohol-related problems may be explained by the assumption that perceived life satisfaction may affect an individual's use of alcohol and other substances through a regulation process (53). The main argument behind this assumption is that regular use of substances is associated with low life satisfaction (53). Other evidence that support this assumption is studies that have found that some individuals respond to a decline in life satisfaction by drinking alcohol in an attempt to improve their life satisfaction (54, 55). It is also likely that the high alcohol consumption can reduce students' life satisfaction through the negative consequences and problems related to their alcohol use (35, 36, 56, 57). For instance, high alcohol consumption among university students can lead to diminished cognitive abilities, impaired motor skills and weakened academic performances (34). It is likely that multiple hypotheses operate at the same time, and the present study cannot rule out that there is a reciprocal interaction between satisfaction with life and harmful alcohol use.

### Association Between Mental Health Problems and Potential Alcohol-Related Problems

The present study also investigated the association between mental health problems and potential alcohol-related problems among students. The results indicated that increased levels of mental health problems heightened the risk of reporting potential alcohol-related problems. The results are in line with other studies. Tembo et al. (25) used AUDIT and divided potential alcohol-related problems into four risk levels: low risk (<8), risky or hazardous (8–15), harmful (16–19) and high risk (≥20). They found that students with moderate or higher levels of mental health problems were 1.3 times more likely to report risky or harmful alcohol use, compared with those with lower levels of mental health problems. Martinez et al. (58) also used AUDIT, and found that students with a history of mental health problems and no affiliation in a social group (fraternity or sorority membership) had increased risk for reporting alcohol-related problems during their freshman year.

Similar results have also been found when mental health problems have been treated as the dependent variable. Seo et al. (27) found in their study with South-Korean students, that both AUDIT-scores and rate of risky alcohol use (males: AUDIT ≥12, females: AUDIT ≥8) were higher in the depressed group, compared with the non-depressed group. In addition, Sæther et al. (17) found that students who reported risky alcohol use (AUDIT 8-17) had slightly reduced life satisfaction and more mental health problems compared with the students who reported low risk alcohol use. The students who reported harmful alcohol use (AUDIT ≥18) had both low life satisfaction and more mental health problems compared with the other students.

#### The Shape of the Association Between Mental Health Problems and Potential Alcohol-Related Problems

In the present study we observed curvilinear associations between mental health problems and potential alcohol-related problems. There was an inverse *U*-shaped association between mental health problems and risky alcohol use, after the removal of 2.5% of the responses on each end of the HSCL-25 scale. The same curvilinear association was found for both female and male students, although the curvature was more pronounced among females. The curvilinear model indicated that students with moderate levels of mental health problems had the highest probability of reporting risky alcohol use. Further, it was found a curvilinear association between mental health problems and harmful alcohol use. The curvilinear association was similar across both genders, but the increase was more pronounced among male students with higher HSCL mean scores. Across all samples (full sample, females, and males), the students with high levels of mental health problems had the highest probability of belonging to the group. High levels of mental health problems are associated with many negative consequences, like isolation, poor study progress, fewer personal relationships and reduced general health (1, 59). Taken together with the assumption that the harmful alcohol use may reflect problem drinking, the results could indicate a comorbidity, where mental illnesses and substance use disorders occur at the same time (21).

The association between mental health problems and potential alcohol-related problems can be explained by multiple hypotheses. Firstly, the negative consequences associated with a harmful alcohol use, such as poor mental and physical health, and loss of work and social relations, may cause or worsen mental health problems (18, 60). Secondly, the association may be a result of self-medication. According to this hypothesis, it is possible that students use alcohol to alleviate or reduce uncomfortable affective states caused by mental health problems (61, 62). Thirdly, mental health problems and alcohol misuse may have a negative reciprocal effect on each other, through the creation of a negative cycle (63). In this way, increased mental health problems and alcohol use may reinforce each other. It is likely that multiple hypotheses operate at the same time. Finally, the association could be explained by for instance trauma exposure. Symptoms of PTSD and trauma among students enrolled in higher education is common (64), and is associated with an increased alcohol use (65).

Despite finding curvilinear associations, our results differ from other studies that also have investigated the shape of the relationship. Skogen et al. (24) found a *U*-shaped curve, where abstainers and heavy drinkers had the highest risk of reporting anxiety and depression. In contrast, Caldwell et al. (31) found a *J*-shaped curve, where heavy drinkers had a higher risk of reporting symptoms of depression. Peltzer and Pengpid (32) also found an inverse *U*-shaped association between alcohol consumption and depression, but contrary to the others it was the students with low alcohol consumption that had an increased risk. It is unclear whether the different results between the present study and the other studies reflects actual differences or is caused by methodological differences. For instance, Skogen et al. (24), Caldwell et al. (31) and Peltzer and Pengpid (32) measured alcohol consumption, while the present study used AUDIT as a measure of risky and harmful alcohol use. The current study also used HSCL-25 to measure mental health problems, while Skogen et al. (24) and Caldwell et al. (31) used different scales, and Peltzer and Pengpid (32) only measured depression.

### Methodological Considerations

This study has several strengths. First, SHoT 2018 is a survey with a very large sample size (*N* = 48 886), and the dataset had little missing data. Second, all variables of interest were assessed using well-validated instruments. Some study limitations should also be noted. First, there was a low response rate (31%), which may increase the chance of selection bias (66, 67). Non-participants have generally poorer health than participants, which may lead to an underrepresentation of mental health problems and potential alcohol-related problems and overrepresentation of satisfaction with life in the current sample (68). However, the impact of non-participation may be less marked for studies investigating associations (68–70). Secondly, the cross-sectional design limits the ability to address direction of causality in the relationship between satisfaction with life, mental health problems and potential alcohol-related problems. Thirdly, AUDIT was originally developed to screen for alcohol-related problems in the adult population (42, 43). It is therefore unknown if the cut-offs for alcohol-related problems is suitable for the student population. In addition, the present study used the same cut-off for females and males (≥8), which some indicate may be too high for females (43). We are, however, not aware of validated cut-points for use in a student population. It is possible that other cut-off points may be more suitable for a student population in general and for this study population in particular, but we are not able to assess this based on the available data from our study. Finally, the present study only focused on alcohol use and did not measure other substances. Symptoms of depression have been associated with use of cannabis, certain “harder” drugs and tobacco among students (4), and both alcohol, drugs and tobacco are in turn associated with increased rates of other substance use problems (71).

## Implications and Conclusion

The present study confirms the high rates of potential alcohol-related problems among Norwegian university and college students (44, 72). The findings suggest that there is a need to increase awareness of mental health issues and responsible alcohol consumption among students, and educational institutions may be an ideal setting for such efforts. By examining the shape of associations between satisfaction with life, mental health problems and potential alcohol-related problems, we were able to look more closely at the relationship between the constructs. Of particular interest, we found that the probability of reporting risky and harmful alcohol use did not necessarily increase in a linear fashion with regard to satisfaction with life and mental health problems. These finding should be taken into consideration in the development of interventions. Students with moderate satisfaction with life and moderate levels of mental health problems had a high probability of belonging to the risky drinking group. For harmful alcohol use, we found that students with low satisfaction with life and high levels of mental health problems had the highest probability of belonging to the group. This seems like a group that is more at risk of experiencing negative consequences than the students with risky alcohol use. Thus, these findings suggest that there might be different target groups depending of the aim of the interventions, and could inform the development of more tailored programs to reduce alcohol consumption among various student groups. The present study confirms previous research that state that there exists a non-linear relationship between mental health problems and alcohol consumption (24, 30, 31). In general, the findings of different shapes of associations may indicate that alcohol use is experienced as something positive and social for some students, but negative and risky for others. Future research should investigate the consequences of scoring moderate levels of satisfaction with life and mental health problems, and investigate whether risky alcohol use reflects social drinking among students.

## Data Availability Statement

The SHoT dataset is administrated by the NIPH. Approval from a Norwegian regional committee for medical and health research ethics [https://helseforskning.etikkom.no] is a pre requirement. Guidelines for access to SHoT data are found at [https://www.fhi.no/en/more/access-to-data].

## Ethics Statement

The studies involving human participants were reviewed and approved by The Regional Committee for Medical and Health Research Ethics in Western Norway (no. 2017/1176). The participants provided their written informed consent to participate in this study.

## Author Contributions

JS conceived of the study. PJ performed the data analyses in close cooperation with JS and wrote the first draft of the paper. JS, EH, and BS critically revised it for important intellectual content. All authors have read and approved the paper for submission.

## Conflict of Interest

The authors declare that the research was conducted in the absence of any commercial or financial relationships that could be construed as a potential conflict of interest.
