# Supplementary material for: Satisfaction With Life, Mental Health Problems and Potential Alcohol-Related Problems Among Norwegian University Students
Source: Front Psychiatry. 2021 Feb 9;12:578180. doi: 10.3389/fpsyt.2021.578180 (PMC7900511; doi:10.3389/fpsyt.2021.578180)

Appendix: Satisfaction with life, mental health problems and potential alcohol-related problems among Norwegian university students

Gender-specific graphical presentation of linear, quadratic and cubic models for the associations between satisfaction with life, mental health problems and potential alcohol-related problems.

All analyses adjusted for age and marital status.

Figure A1: Associations between satisfaction with life and potential alcohol-related problems. Gender-specific analysis.

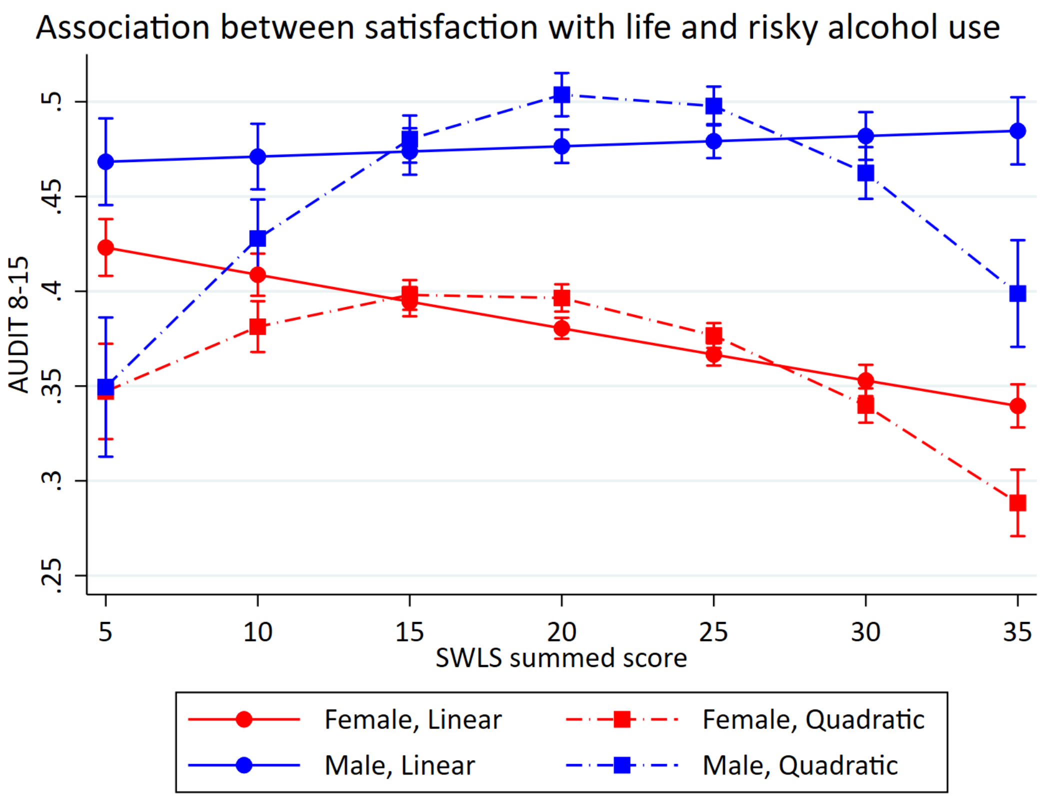

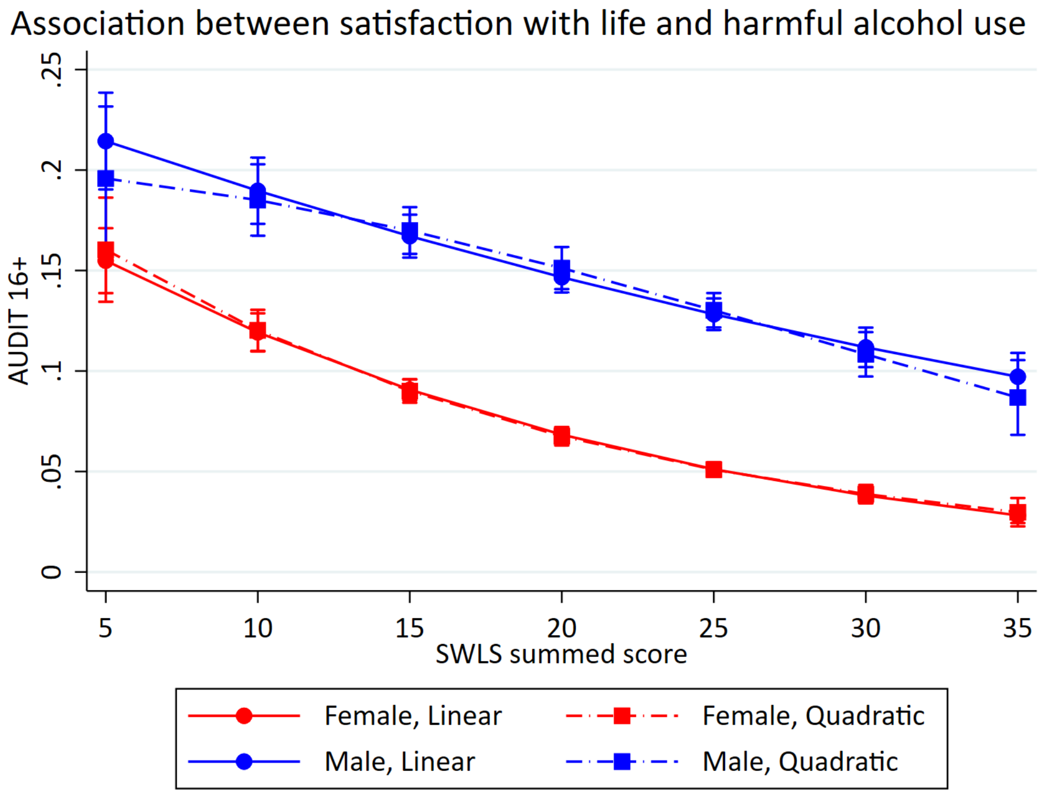

Figure A2: Association between mental health problems and potential alcohol-related problems. Gender-specific analysis.

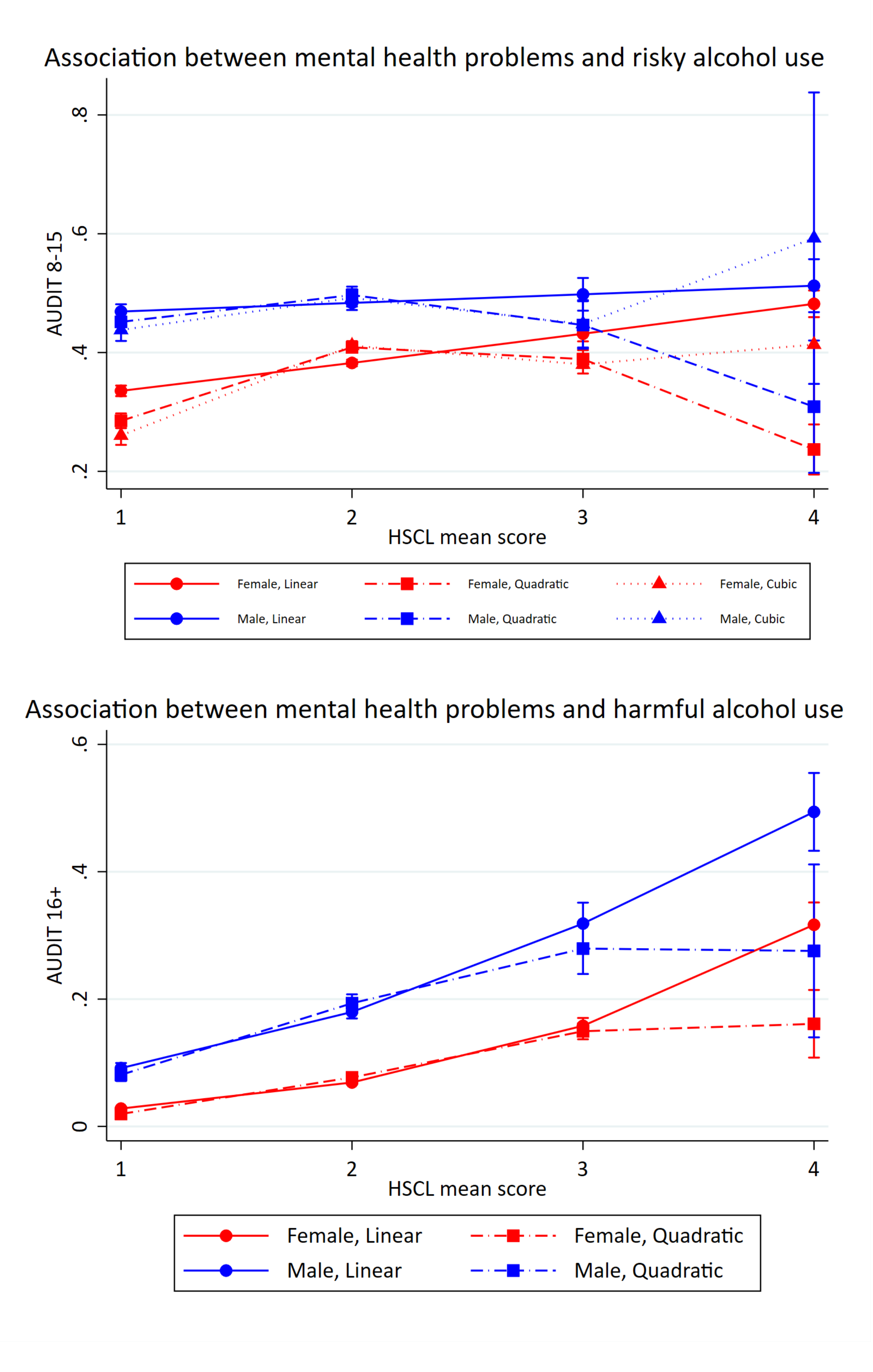

Figure A3: Association between mental health problems and risky alcohol use (after the removal of 2.5% in each end of HSCL-25 scale). Gender-specific analysis.

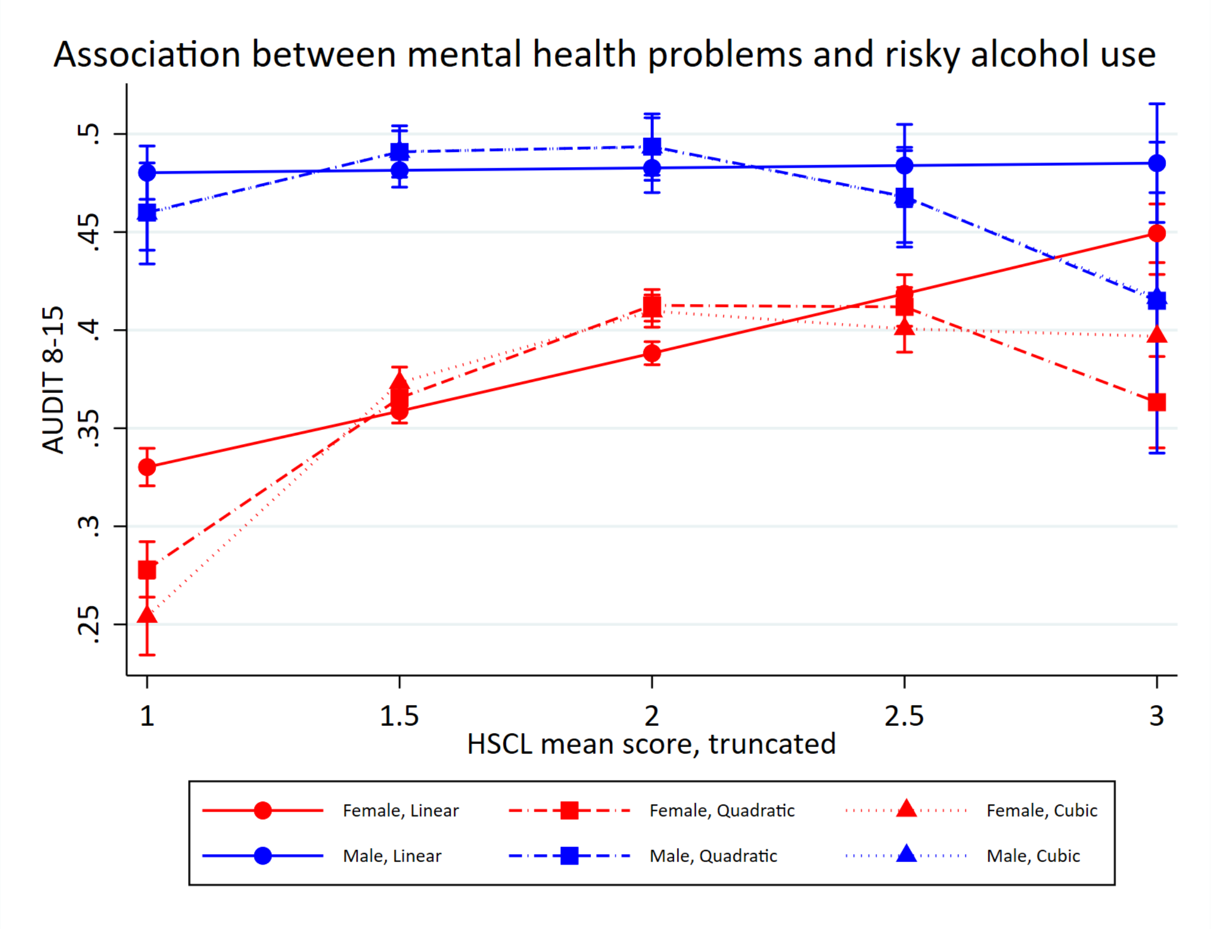

Supplement: Supplementary file 1 [file Image_1.pdf]
